# A Multistrategy Optimization Improved Artificial Bee Colony Algorithm

**DOI:** 10.1155/2014/129483

**Published:** 2014-04-03

**Authors:** Wen Liu

**Affiliations:** ^1^The School of Computer Science and Technology, Dalian University of Technology, Dalian, China; ^2^Department of Electrical Engineering, Xinjiang Institute of Engineering, Tianjin Road, No. 176, Urumqi 830011, China

## Abstract

Being prone to the shortcomings of premature and slow convergence rate of artificial bee colony algorithm, an improved algorithm was proposed. Chaotic reverse learning strategies were used to initialize swarm in order to improve the global search ability of the algorithm and keep the diversity of the algorithm; the similarity degree of individuals of the population was used to characterize the diversity of population; population diversity measure was set as an indicator to dynamically and adaptively adjust the nectar position; the premature and local convergence were avoided effectively; dual population search mechanism was introduced to the search stage of algorithm; the parallel search of dual population considerably improved the convergence rate. Through simulation experiments of 10 standard testing functions and compared with other algorithms, the results showed that the improved algorithm had faster convergence rate and the capacity of jumping out of local optimum faster.

## 1. Introduction

The artificial bee colony algorithm is a new heuristic optimization algorithm proposed in recent years by Karaboga [1]. References [2, 3] pointed out that by comparing the performance of optimization of differential evolution algorithm [4] and the particle swarm algorithm [5], ABC algorithm obtained more favorable test results and is one of the most outstanding function optimization methods, which has become a hot topic at the forefront of domestic and foreign heuristic algorithm researches.

However, similar to other intelligent algorithms, the standard ABC algorithm also had disadvantages of easily prematurely falling into local optima and slow convergence rate in later stage. In this regard, a number of scholars made corresponding improvement. Reference [6] proposed an artificial bee colony algorithm solving the problem of minimum spanning tree; [7] proposed an improved algorithm where combining particle swarm algorithm, to some extent, accelerated the local convergence rate of algorithm; and [8] proposed an improved algorithm combining differential evolution algorithm and artificial bee colony algorithm, which effectively improved the searching accuracy and the convergence rate of the algorithm to some extent. However, improved algorithms above did not effectively improve the convergence rate and avoid premature convergence problem at the same time.

In order to overcome premature to improve the convergence speed and optimal accuracy, this paper proposes a new improved artificial bee colony algorithm. First, a chaos reverse learning strategy was proposed and introduced into the initialization phase of artificial bee colony algorithm, making the initial population uniformly distributed in the search space, in order to improve the quality of population solution, thus speeding up the global convergence rate of the algorithm. Secondly, according to the idea of separate optimization and survival of the fittest, two populations was filter optimized by using dual population structure, thus the optimization process was accelerated in the case of maintaining the population diversity. The introduction of the dynamic adaptive idea to the algorithm and the improvement of nectar update formula based on the comparison of population diversity measurement values, the problem of algorithm falling into a local optimum was solved. The simulation results of optimization of 10 standard test functions that were widely used showed that, comparing with the existing two artificial bee colony algorithms, the proposed algorithm had better optimization accuracy, convergence rate, and robustness.

## 2. Algorithm Description

### 2.1. Artificial Bee Colony Algorithm

Artificial bee colony algorithm uses simulating the mechanism of bees collecting nectar to achieve the optimization processing function. In artificial bee colony algorithm, the bees are divided into three categories, that is, employed bees, onlookers, and scouts [9]. The main task of employed bees and onlookers is to search and mine nectar, and scouts are used to search and compare nectar in order to avoid few nectar species. The location and quantity of the nectar are the solution of function optimization problem and corresponding function value. The process of searching optimal nectar is as follows: employed bees find nectar and memorize and search for new nectar in the vicinity of each nectar; at the same time employed bees release information that is proportional to the mass of marked nectar to attract onlookers. Onlookers select the appropriate marked nectar under some mechanism and search for new nectar source in the vicinity and compare with selected nectar. Select excellent quality nectar as final marked nectar, looking for the best nectar in repeated cycles. If during the process of collecting nectar, after several searches, nectar is unchanged, then corresponding employed bees are changed into scouts and they randomly search for new nectar [9–11]. The function optimization problem can be expressed as follows:

(1) min⁡ fit=fit(x), x=(x1,x2,…,xn)∈S,  S=[xi,L,xi,H],

where fit represents the objective function, **x** is an *n*-dimensional variable, and [*x*_*i*,*L*_, *x*_*i*,*H*_] is the corresponding upper and lower bounds of the *i*th dimensional variable. Set the number of nectar, employed bees, and onlookers to be *N* in the ABC algorithm. The specific steps of ABC algorithm are as follows.

**Step 1**

2*N* nectar positions are generated randomly by the following formula:

(2) $$\begin{array}{c} V_{ij}=x_{j,L}+\text{rand}\times \left(x_{j,H}-x_{j,L}\right), \end{array}$$

where *V*_*ij*_ is the corresponding search position of *i*th bee in *j*th dimension; *x*_*j*,*L*_, *x*_*j*,*H*_ are upper and lower bounds of the *j*th dimensional variables; select *N* positions with low fitness values as the position of nectar.

**Step 2**

Employed bees search and update nectar in the vicinity of nectar according to the following:

(3) $$\begin{array}{c} V_{ij}=x_{ij}+r_{ij}\times \left(x_{ij}-x_{kj}\right), \end{array}$$

where *V*_*ij*_ is the position of new nectar, *x*_*ij*_ is the *j*th dimensional position of nectar *i*, *x*_*kj*_ is *j*th dimensional position of randomly selected nectar *k*, and *k* ≠ *i*, *r*_*ij*_ is a random number of [−1,1].

**Step 3**

Comparing the pros and cons of before and after nectar, replace the previous nectar, if after searching nectar is superior to previous nectar.

**Step 4**

According to the way of roulette and nectar information released by employed bees, onlookers select nectar, the selection probability of onlookers is as follows:

(4) $$\begin{array}{c} P_{i}=\frac{\text{fit}\left(x_{i}\right)}{\sum_{i=1}^{N}\text{fit}\left(x_{i}\right)}. \end{array}$$

**Step 5**

Onlookers search for new nectar in accordance with (3), and compared with the nectar quantity searched by employed bees, Set the N position of more nectar as the position of employed bees; the rest is position of onlookers.

**Step 6**

If some nectar is unchanged after limit cycles, the nectar is given up, corresponding employed bees are turned into onlookers, and new nectar is randomly generated according to (2).

**Step 7**

Record location of best nectar source and return to Step 2 until the termination condition is met.

### 2.2. Particle Swarm Algorithm

Mathematical description of the particle swarm intelligence algorithm [5] is as follows. Suppose in a *D*-dimensional target space, *N* particles with potential problem solutions composed a group, where *i*th particle is represented as a *D*-dimensional vector, *X*_*i*_ = [*X*_*i*1_,*X*_*i*2_,…,*X*_*iD*_]^*T*^(*i* = 1,2, …, *N*); position of the *i*th particle in *D*-dimensional search space is *X*_*i*_; flight speed is *V*_*i*_; *P*_*i*_ is the personal best position searched by *i*th particle so far; and remember *P*_*g*_ is the global optimal position searched by particle swarm so far; in each iteration, particles update speed and position in accordance with

(5) $$\begin{array}{c} V_{i}^{t+1}=wV_{i}^{t}+c_{1}r_{1}\left(P_{i}^{t}-X_{i}^{t}\right)+c_{2}r_{2}\left(P_{g}^{t}-X_{i}^{t}\right),\\ X_{i}^{t+1}=X_{i}^{t}+V_{i}^{t+1}, \end{array}$$

where, *i* = 1,2, 3,…, *N*, *t* is the number of iterations; *w* is the inertia coefficient; *c*_1_, *c*_2_ are learning factors and suitable *c*_1_, *c*_2_ can speed up convergence and not easily fall into local optimum; and *r*_1_, *r*_2_ are random numbers in [0,1]. Particles find *P*_*g*_ which is the global optimal solution via constantly learning and updating [5, 12–14]. Particle swarm algorithm is applied to the positioning phase of the proposed algorithm; the main steps are as follows.

**Step 1**

Determine parameters: the number of particles *N*, the inertia factor *w*, and the number of iterations *t*.

**Step 2**

Randomly generate a population of *N* particles.

**Step 3**

Update velocity and position of particle using (5).

**Step 4**

Global optimal solution *P*_*g*_ is obtained by comparing and calculating the fitness function values; solution is the coordinates of unknown node.

**Step 5**

Determine whether the condition of the loop termination was met, if it was, record coordinates of unknown node, otherwise return to Step 3.

## 3. Improved Artificial Bee Colony Algorithm

### 3.1. Chaos Reverse Learning Strategies

Population initialization is particularly important in intelligent algorithm, because initialization quality directly affects the algorithm global convergence speed and the corresponding solution quality. Under normal circumstances, due to the lack of a priori information, random initialization is often used to generate the initial solution of algorithm. Reference [15] proposed a chaotic initialization method in the process of researching particle swarm algorithm, while [16] proposed initialized method of reverse learning. On this basis, this paper proposed a chaotic reverse learning strategy by combining these two initialization methods, and the strategy was used to initialize ABC algorithm; concrete steps are as follows.

**Figure 1:**
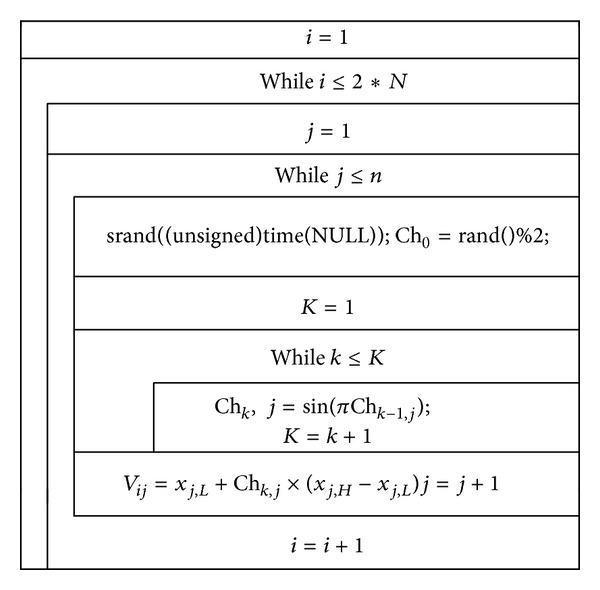
The *N*-*S* chart of chaotic phase.

**Figure 2:**
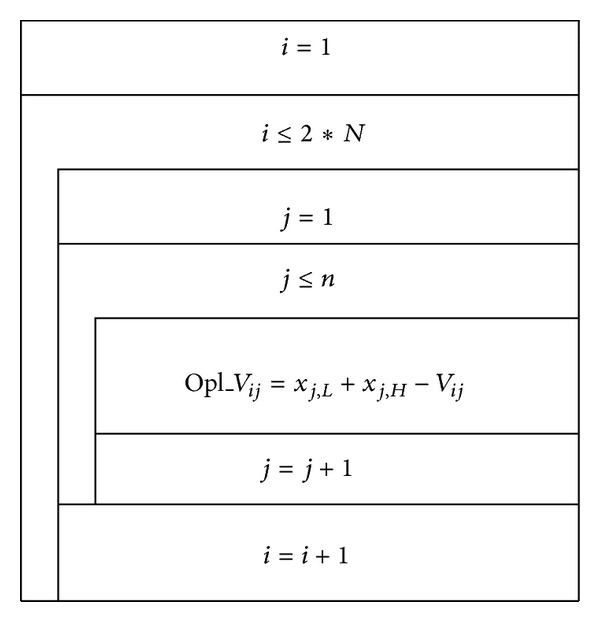
The *N*-*S* chart of reverse learning phase.

1. Set maximum chaotic iteration step *K* ≥ 400 and the population size 2*N*. The *N*-*S* charts of chaotic phase and reverse learning phase are given in Figures 1 and 2.
2. Select 2*N* best fitness value particles as the initial bee swarm from {*V*(2*N*) ∪ Opl_*V*(2*N*)}.

### 3.2. Dynamic Self-Adaptive Nectar Update Strategy

In the process of searching nectar source, employed bees often choose nectar source with more nectar quantity, but when many employed bees select the same nectar, this information amount of nectar will increase in vain, which causes too many employed bees to concentrate on one nectar source, causing blockage or stagnation. When solving the optimization problem, this will manifest premature and local convergence [17–20]. In order to solve this problem, a new dynamic self-adaptive nectar update strategy was proposed. This strategy introduced the concept of population diversity measurement and it was used to the redefinition of nectar update formula, in order to improve the algorithm search capabilities.

Reference [21] pointed out that the difference between the average particle distance and particle fitness was commonly used to indicate the population diversity. On the basis of analyzing disadvantages of this approach, the similarity degree of the individuals in population was used to characterize the population diversity, which was introduced to the updated nectar formula that is formula (3). Let individual number of ABC algorithm be 2*N*, and the *j*th individual of *i*th generation bee colony **x**_*i*_ is **x**_*i*,*j*_ = (*x*_*i*,*j*(1)_, *x*_*i*,*j*(2)_,…, *x*_*i*,*j*(*n*)_), where *n* is the number of nectar solution dimensions; the *j*th individual successive dynasties nectar optimum position is **y**_*j*_ = (*y*_*j*(1)_, *y*_*j*(2)_,…, *y*_*j*(*n*)_). Combine individual nectar position and successive dynasties optimum position together, referred to as **Z**_*j*_ = (*x*_*i*,*j*(1)_, *x*_*i*,*j*(2)_,…, *x*_*i*,*j*(*n*)_); all individuals **Z**_*j*_ in bee colony can be composed of a matrix **Z** of 2*N* × 2*n* order, normalization process **Z**, and matrix **Z** of 2*N* × 2*n* order can be obtained as

(6) $$\begin{array}{c} Z^{\prime }=\frac{Z_{uv}-\min_{1\leq g\leq 2N,1\leq l\leq 2n}Z_{gl}}{\max_{1\leq g\leq 2N,1\leq l\leq 2n}Z_{gl}-\min_{1\leq g\leq 2N,1\leq l\leq 2n}Z_{gl}}, \end{array}$$

where 1 ≤ *u*≦2*N*, 1 ≤ *v* ≤ 2*n*, and each row vector can be seen as a fuzzy set, expressed as membership degrees of the nectar current location and successive position of each component searched by *j*th employed bee or onlooker; any similarity degree of two **Z**_*u*_′, **Z**_*v*_′ can be expressed by nearness; that is,

(7) $$\begin{array}{c} L\left(u,v\right)=1-\frac{1}{2n}\overset{2n}{\underset{t=1}{\sum }}\left|Z_{u,t}^{\prime }-Z_{v,t}^{\prime }\right|. \end{array}$$

Bees Diversity Measurement *F* can be expressed by population average nearness

(8) $$\begin{array}{c} F=\frac{2\sum_{u=1}^{2N-1}\sum_{v=u+1}^{2N}L\left(u,v\right)}{2N\left(2N-1\right)}. \end{array}$$

*F* ≤ 1 is obtained by 0 ≤ *L*(*u*, *v*) ≤ 1; if individuals in bee colony are identical, then the diversity is the worst; *F* is the maximum value 1.

In the update nectar formula of ABC algorithm, since *r*_*ij*_ is randomly generated numerical value in [−1,1], the relationship between nectar source and the diversity of employed bees and bee colony is ignored. Therefore, let *r*_*ij*_ therefore, let adjustment formula of *r*_*ij*_ be:

(9) $$\begin{array}{c} r_{ij}\left(k\right)=\frac{1}{\left(a-bF\left(k-1\right)\right)}, \end{array}$$

where *r*_*ij*_(*k*) is the update coefficient of *k*th generation bee colony, *F*(*k* − 1) is diversity measurement of (*k* − 1)th bee colony, and *a*, *b* are constants. Update formula of improved nectar is

(10) $$\begin{array}{c} V_{ij}\left(k\right)=x_{ij}+r_{ij}\left(k\right)\times \left(x_{ij}-x_{kj}\right). \end{array}$$

### 3.3. Dual Population Search Strategy

Since the update methods of ABC and PSO Algorithm individual, as well as different optimization strategies, the effect of optimization also varies. In order to improve the population diversity of ABC algorithm and accelerate the speed of algorithm searching for optimal solution, inspired by reference [8, 22], taking into consideration the advantages of particle swarm algorithm which has a simple structure, easy to implement, few parameters, and fast algorithm convergence rate in early stage, the advantages of this algorithm and ABC algorithm are combined to propose a dual population search strategy. Main ideas of the strategy are to randomly divide the population into two groups, each group using different optimization strategies to find optimal solution. Better solution is selected as the algorithm optimal solution after comparison. The specific process is as follows.

**Step 1**

Initialize population behaviors using chaos reverse learning strategy mentioned above.

**Step 2**

Initialized population is randomly divided into two groups; one group uses improved ABC algorithm mentioned above, nectar update using formula (10); another group uses particle swarm algorithm, individual update using formula (5).

**Step 3**

Two kinds of populations are searching for optimal solution in accordance with their respective search strategy under algorithm termination condition. And based on the idea of survival of the fittest, respective proceeds in accordance with the respective optimal solutions are compared, and position of the better solution is recorded.

Diversity of the population is ensured by mixing two populations and two populations parallel searching; at the same time, algorithm convergence rate is improved to a large extent; the algorithm has a higher convergence rate in reasonable computational complexity.

## 4. Convergence Analysis

IMABC algorithm in this paper determines convergence according to methods given in the literature [23–25].

### 4.1. Convergence Criteria

If the result of the iteration of optimization problem {*A*, *f*} is *x*_*k*_, then the next iteration is *x*_*k*+1_ = *Q*(*x*_*k*_, *η*), of which *A* is solution space, *f* is fitness function, and *η* is the solution which the algorithm has found. The function

(11) $$\begin{array}{c} R_{\delta ,Z^{+}}=\{\begin{array}{cc} \left\{x\in A\;|\;f\left(x\right)<\beta +\delta \right\}, & \beta <\left|\infty \right|,\;\;\delta >0;\\ \left\{x\in A\;|\;f\left(x\right)<-Z^{+}\right\}, & \beta =-\infty ; \end{array} \end{array}$$

is defined as the optimal area; if the algorithm finds a point in *R*_*δ*,*Z*^+^_, then the algorithm can be considered to find the optimal algorithm or approximate optimal solution.

**Condition 1**

*f*(*Q*(*x*, *η*)) ≤ *f*(*x*); if *η* ∈ *A*, then*f*(*Q*(*x*, *η*)) ≤ *f*(*η*). If the algorithm satisfies this condition, it can be stated that fitness is nonincremental.

**Condition 2**

For any Borel subset *B* of *A*, if in the set *B* the Lebesgue measure *v*[*B*] > 0, then ∏_*k*=0_^*∞*^(1 − *u*_*k*_[*B*]) = 0. If the algorithm satisfies the condition, it can be stated that after bee colony unlimitedly searches optimization, the probability of global optimum that cannot be found is 0.

**Theorem 1**

Set the function *f* as measurable; *A* is a measurable subset of *R* ^*n*^, algorithm *Q* satisfies Conditions 1 and 2, and {*x*_*k*_}_*k*=0_^*∞*^ is the solution sequence generated by algorithm *Q*; there lim⁡_*k*→*∞*_⁡*P*(*x*_*k*_ ∈ *R*_*δ*,*Z*^+^_) = 1.

### 4.2. Algorithm Convergence Analysis

**Lemma 2**

IMABC algorithm meets Condition 1.

**Proof**

The algorithm uses chaotic reverse learning strategies to initialize population, double-population search is conducted in each iteration, and the optimal value is saved; that is,

(12) $$\begin{array}{c} H\left(P_{g,t}V_{i,t}x_{i}\right)=\{\begin{array}{cc} P_{g,t}x_{i}, & f\left(V_{i,t}\right)\leq f\left(x_{i}\right),\\ V_{i,t}x_{i}, & f\left(V_{i,t}\right)>f\left(x_{i}\right). \end{array} \end{array}$$

Condition 1 is met.

**Definition 3**

Assuming optimal solution is *g*_best_; optimal solution set is defined as *G* = {*s* = (*X*) | *f*(*X*) = *f*(*g*_best_), *s* ∈ *S*}.

**Theorem 4 (see [13, 23–25])**

In the algorithm, for bee colony state sequence {*s*(*t*); *t* ≥ 0}, set *G* as a closed set in state space *S*.

**Proof**

Set ∀*s*_*i*_ ∈ *G*, ∀*s*_*j*_ ∉ *G*; for any transfer step length *l*, *l* ≥ 1, the probability *P*_*s*_*i*_,*s*_*j*__^*l*^of bee colony state transferred from *s*_*i*_ to *s*_*j*_ by *l* steps can be obtained by bee colony algorithm

(13) Psi,sjl=∑sr1∈s⋯∑srl−1∈sP(Ts(si)=sr1)       ×P(Ts(sr1)=sr2)⋯P(Ts(srl−1)=sj),

where *P*(*T*_*s*_(*s*_*r*_*c*−1__) = *s*_*r*_*c*__) is the probability of bee colony state transferred from *s*_*c*−1_ to *s*_*c*_, 1 ≤ *c* ≤ *l*, and the probability is determined by transition probability of each bee; that is, *P*(*T*_*s*_(*s*_*r*_*c*−1__) = *s*_*r*_*c*__) = ∏_*m*=1_^*SN*^ *p*(*T*_*s*_(*X*_*im*_) = *X*_*jm*_). *SN* is the number of bees. *P*(*T*_*s*_(*s*_*r*_*c*−1__) = *s*_*r*_*c*__) exists in each expression in (13), since *s*_*r*_*c*−1__ ∈ *G*, *s*_*r*_*c*__ ∉ *G*, *f*(*X*_*c*_) > *f*(*X*_*c*−1_) = *f*(*g*_best_) = inf⁡(*f*(*a*)), *a* ∈ *A*; at least there exists *P*(*T*_*s*_(*s*_*r*_*c*−1__) = *s*_*r*_*c*__) = 0, *P*_*s*_*i*_,*s*_*j*__^*l*^ = 0, so set *G* as a closed set in state space *S*.

**Theorem 5 (see [23–25])**

Bee colony state space *S* does not have a nonempty closed set *M*, making *M*∩*G* = *ϕ*.

**Proof**

Assume that there exists a nonempty closed set *M* and *M*∩*G* = *ϕ*. Set *s*_*i*_ = (*g*_best_, *g*_best_,…, *g*_best_) ∈ *G*, ∀*s*_*j*_ = (*x*_*j*1_, *x*_*j*2_,…, *x*_*jd*_) ∈ *M*; since*f*(*X*_*jc*_) > *f*(*g*_best_), after a finite number of iterations, the probability of scouter transferred from *X*_*i*_ to *X*_*j*_ is *p*_*sc*_(*T*_*s*_(*X*_*i*_) = *X*_*j*_) > 0. Therefore when the step size is large enough, in expansion *P*_*s*_*i*_,*s*_*j*__^*l*^ there must be a certain product expression greater than 0; that is *P*(*T*_*s*_(*s*_*r*_*c*−*i*__) = *s*_*r*_*c*+*i*+1__) > 0. From (13) in Theorem 4, *P*_*s*_*i*_,*s*_*j*__^*l*^ > 0; *M* is not a closed set, which is contradicted with the question conditions. Therefore, there is no closed set in the state space *S* except *G*.

**Theorem 6 (see [24])**

Assume Markov Chain has a nonempty closed set *E* and there is no other nonempty closed set *O*; let *E*∩*O* = *ϕ*. Therefore, when *j* ∈ *E*, lim⁡_*n*→*∞*_⁡*P*(*X*_*n*_ = *j*) = *π*_*j*_ and when *j* ∉ *E*, lim⁡_*n*→*∞*_⁡*P*(*X*_*n*_ = *j*) = 0 [2].

**Theorem 7**

When bee colony is unlimitedly iterated and optimized, all state sequences are present in the optimal state set *G*.  Theorem 7 is established which can be drawn from Theorems 4–6.

**Lemma 8**

Algorithm satisfies Condition 2.

**Proof**

By Theorem 7, after bee colony is unlimitedly optimized, the probability of no global optimum is 0; then there is ∏_*k*=0_^*∞*^(1 − *u*_*k*_[*B*]) = 0.

**Theorem 9**

IMABC algorithm converges to global optimum.

As the bee colony algorithm satisfies Conditions 1 and 2, the algorithm can be obtained and converged to the global optimum by Theorem 1. The algorithm in this paper uses two optimization algorithms and the optimal solutions obtained from two populations are recorded; the two populations are independent. As long as the global optimal solution probability of one of the optimization algorithms converges to 1, the global optimal solution probability of the entire algorithm also converges to 1. Therefore, this algorithm is a global convergence algorithm.

## 5. Simulation Experiment and Results Analysis

In order to verify the validity of above analysis and the improved algorithm performance, comparison experiments were done on this improved algorithm (abbreviated as IMABC), traditional ABC algorithm, and improved integration algorithm (abbreviated as PABC) combined by ABC algorithm proposed by [7] and PSO algorithm. In the simulation experiment, 10 test functions [26–29] were selected; $\begin{array}{c} f_{1}\sim f_{8} \end{array}$ are high-dimensional functions, where *f*_1_ and *f*_2_ are unimodal functions; $\begin{array}{c} f_{3}\sim f_{8} \end{array}$ are multimodal functions; and *f*_9_ and *f*_10_ are two-dimensional functions.  Table 1 lists names, dimensions, definitions, ranges, and theory global optimal solutions of these test functions. In the experiments, population sizes of the three algorithms are all 40, *K* is 300, limit is 40, and the corresponding maximum number of iterations is 5000, where parameter settings of dual population strategy particle swarm algorithm are seen in [27]; that is, *c*_1_ = *c*_2_ = 2, *w* = 0.4. For each test function, every algorithm is randomly run 30 times to find the best value, the worst value, average, and standard deviation. The best and worst values reflect the solution quality; average tells the accuracy that algorithm can achieve under a given number of function evaluations, reflecting the algorithm convergence rate; variance reflects the stability and robustness of the algorithm. Results are shown in Table 2.

As can be seen from the data comparison in Table 2, among most standard test functions, whether it is the solution quality or algorithm convergence accuracy and stability, IMABC algorithm has been greatly more improved than PABC algorithm and ABC algorithm. In functions *f*_1_ and *f*_9_, although compared to PABC algorithm IMABC algorithm's minimum, the worst value, average, and variance all slightly increase, significant improvement is achieved when compared to the standard ABC algorithm. In functions *f*_2_, *f*_5_, *f*_6_, and *f*_8_, the improved algorithm is significantly better than the standard ABC algorithms and PABC algorithm in various test results; especially in functions *f*_3_, *f*_4_, *f*_7_, and *f*_10_, IMABC algorithm not only has good test results but also can converge to optimal solution, showing good searching performance.

**Figure 3:**
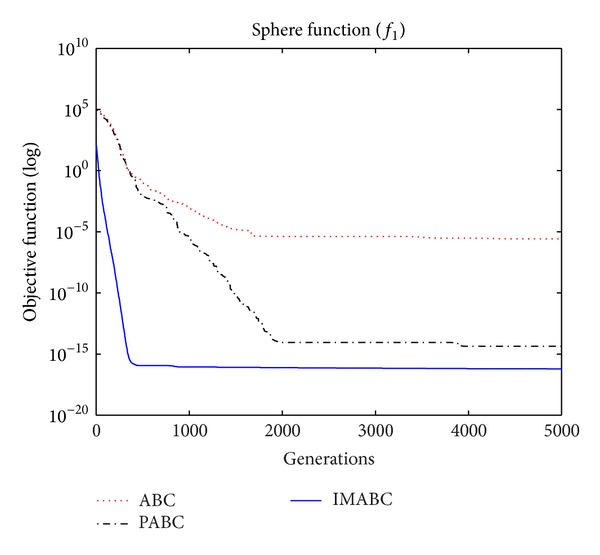
*f*_1_ function convergence performance comparison.

**Figure 4:**
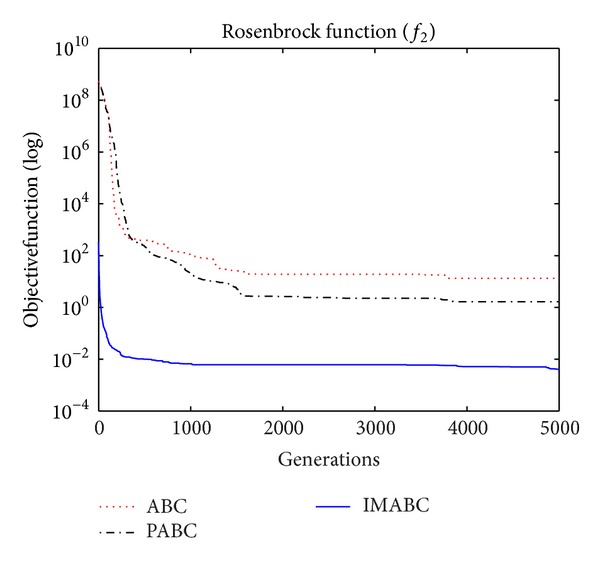
*f*_2_ function convergence performance comparison.

**Figure 5:**
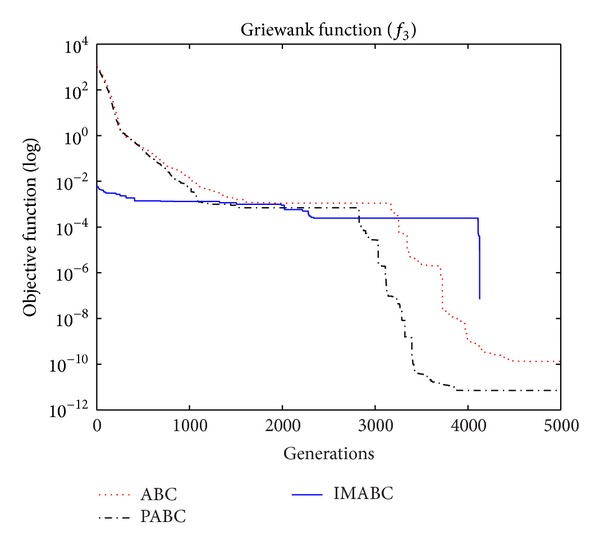
*f*_3_ function convergence performance comparison.

**Figure 6:**
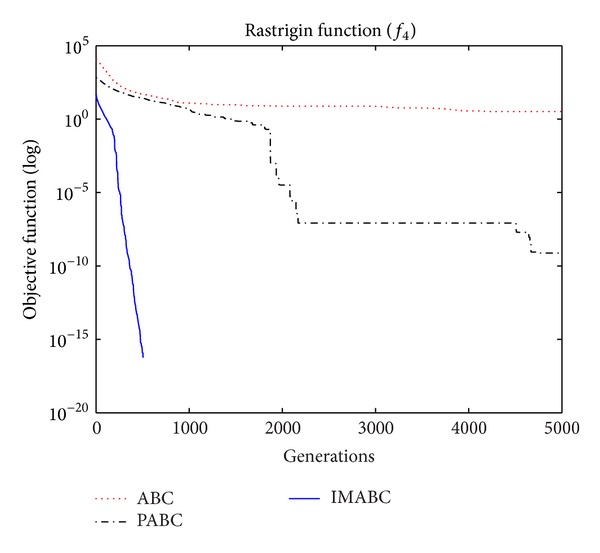
*f*_4_ function convergence performance comparison.

**Figure 7:**
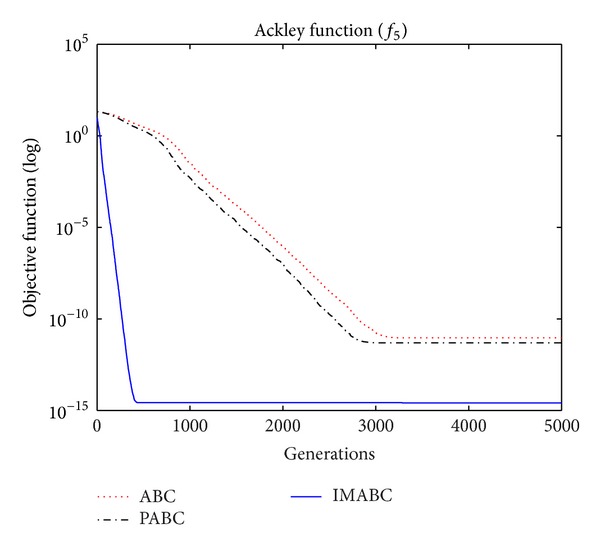
*f*_5_ function convergence performance comparison.

**Figure 8:**
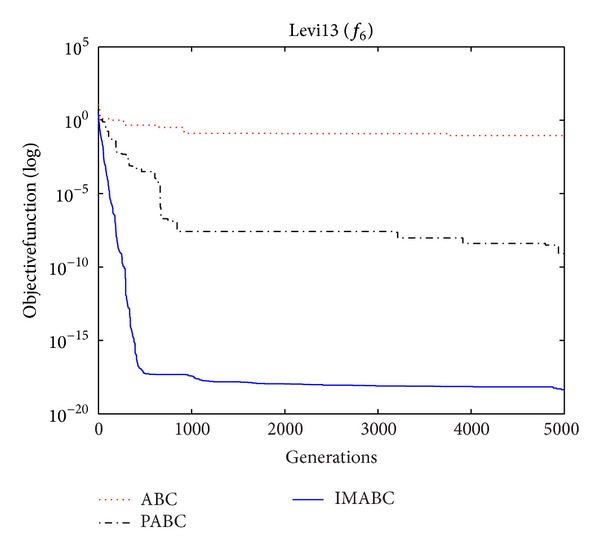
*f*_6_ function convergence performance comparison.

**Figure 9:**
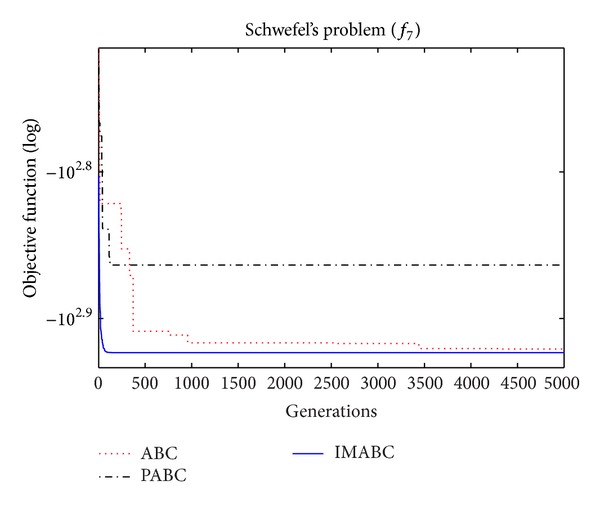
*f*_7_ function convergence performance comparison.

**Figure 10:**
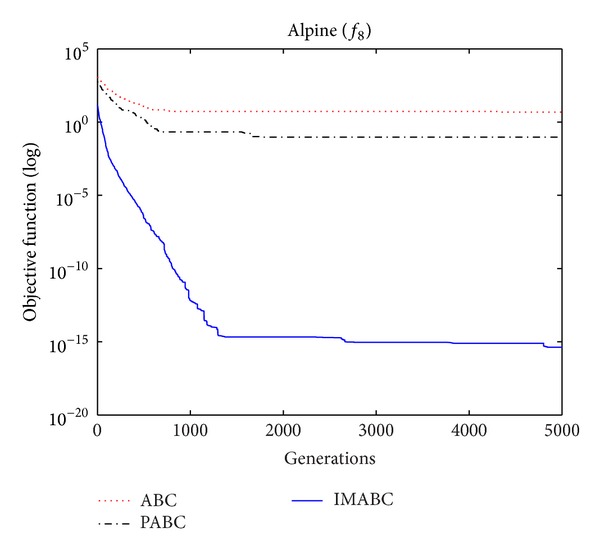
*f*_8_ function convergence performance comparison.

**Figure 11:**
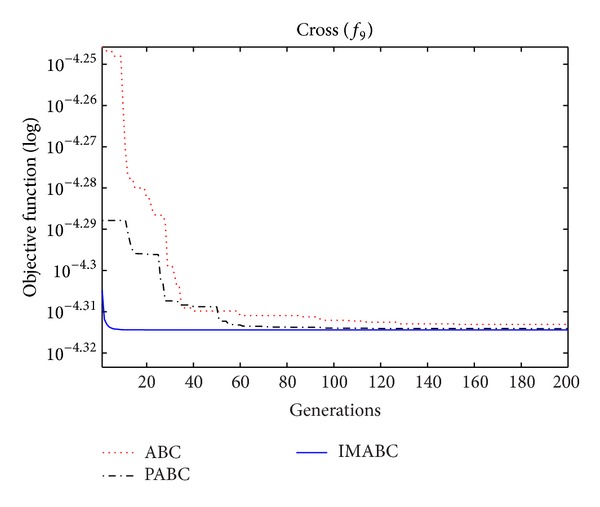
*f*_9_ function convergence performance comparison.

**Figure 12:**
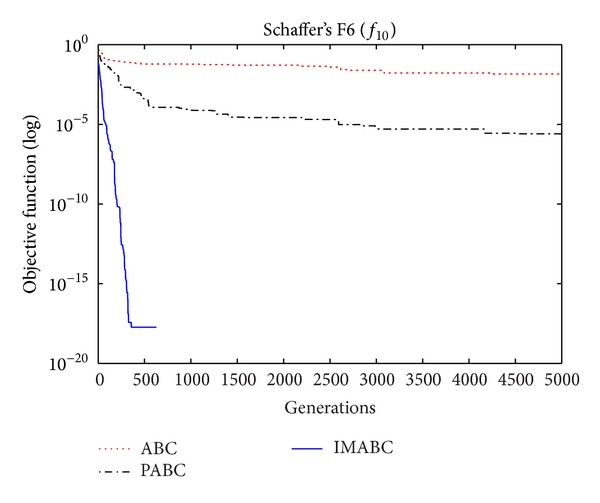
*f*_10_ function convergence performance comparison.

In order to compare algorithm optimization effect more visually, IMABC algorithm, PABC algorithm, and ABC algorithm are compared. Corresponding test function convergence curves are given in Figures 3 to 12. According to the figures, because of the use of a new initialization method and dual population parallel search strategy, as well as the dynamic self-adaptation of nectar location update, the improved Artificial Bee Colony algorithm can jump out of local optimal solution and gradually converge to the global optimal solution when processing multimodal functions, and has a faster convergence rate when in processing unimodal functions and low-dimensional functions. While processing unimodal functions and low-dimensional functions, this improved algorithm has a faster convergence rate. It can be seen from Figures 3, 4, 7, and 8, since standard test function has a high complexity in 50th-dimension, three algorithms are all unable to converge to the optimal solution, but this improved algorithm, compared to ABC algorithm and PABC algorithm, has a faster convergence rate and significantly superior convergence accuracy; it can be seen from Figures 5, 6, 9, and 12 that, compared to the other two algorithms, IMABC algorithm has higher convergence accuracy and can converge to a global optimal solution faster and stabilize. As can be seen from Figure 10, this proposed algorithm gradually approaches function optimal solution with the increase of iterations, although it cannot converge; the extent of approaching and search accuracy are significantly better than the other two algorithms. As can be seen from Figure 11, IMABC algorithm and the other two algorithms are all approaching function optimal solution with the increase of iterations. When the number of iterations is more than 60 times, this algorithm has little difference with PABC algorithm. But when the number of iterations is less than 60 times, it can be seen that IMABC algorithm has higher convergence rate. Therefore, it can be concluded that the overall optimization performance of this proposed IMABC algorithm is superior to the standard ABC algorithm and the PABC improved algorithm proposed in [7].

## 6. Conclusion

In order to avoid falling into local optimum resulting from premature and improve convergence rate of ABC algorithm, an improved artificial bee colony algorithm based on multipolicy optimization was proposed. In order to improve the global search ability and keep the algorithm diversity, improved algorithm proposed a chaotic reverse learning initialization method on the basis of existing research results; in order to avoid the algorithm falling into a local optimum, improved algorithm introduced dual population search mechanism into search phase of the algorithm. Advantages of particle swarm algorithm and standard ABC algorithm were merged; meanwhile, algorithm convergence rate was increased. In addition, in order to improve the algorithm population diversity and global search capability, the concept of population similarity degree was introduced into the improved algorithm, and an indicator of population diversity measure was proposed to dynamic self-adaptive adjustment of the nectar location. Experimental results of 10 standard test functions optimization showed that this proposed algorithm improved more greatly than standard ABC algorithm and PABC in optimization efficiency, optimization performance, and robustness.

Furthermore, this improved algorithm also has certain limitations: though optimization performance is improved, the algorithm complexity is increased to a certain extent. How to ensure algorithm jumping out of local optima and having high convergence rate, at the same time, possessing low algorithm complexity, will be the next step in research.

## Figures and Tables

**Table 1:** Dimension, search space, and optimal value of test functions.

Function	Mathematical representation	Dimension (*D*)	Range of search (*S*)	Theoretical optimum *f*_min⁡_
Sphere (*f*_1_)	$f_{1}(x)=\overset{D}{\underset{i=1}{\sum }}x_{i}^{2}$	50	[−100, 100]^*D*^	0
Rosenbrock (*f*_2_)	$f_{2}\left(x\right)=\overset{D}{\underset{i=1}{\sum }}\left[100{\left(x_{i+1}-x_{i}^{2}\right)}^{2}+{\left(x_{i}-1\right)}^{2}\right]$	50	[−30, 30]^*D*^	0
Griewank (*f*_3_)	$f_{3}(x)=\frac{1}{4000}\overset{D}{\underset{i=1}{\sum }}x_{i}^{2}-\prod_{i=1}^{D}\cos\left(\frac{x_{i}}{\sqrt{i}}\right)+1$	50	[−600, 600]^*D*^	0
Rastrigin (*f*_4_)	$f_{4}(x)=\left(\overset{D}{\underset{i=1}{\sum }}x_{i}^{2}-10\cos\left(2\pi x_{i}\right)+10\right)$	50	[−5.12, 5.12]^*D*^	0
Ackley (*f*_5_)	$\begin{array}{c} f_{5}(x)=-20\exp\left(-0.2\sqrt{\frac{1}{D}\overset{D}{\underset{i=1}{\sum }}x_{i}^{2}}\right)-\exp\left(-0.2\frac{1}{D}\overset{D}{\underset{i=1}{\sum }}\cos\left(2\pi x_{i}\right)\right)+20+e \end{array}$	50	[−30, 30]^*D*^	0
Levi11 (*f*_6_)	$\begin{array}{c} f_{6}(x)=\sin{\left(3\pi x_{1}\right)}^{2}+{\left(x_{1}-1\right)}^{2}(1+\sin{\left(3\pi x_{1}\right)}^{2})+{\left(x_{2}-1\right)}^{2}(1+\sin{\left(2\pi x_{2}\right)}^{2}) \end{array}$	50	[−10, 10]^*D*^	0
Schwefel's problem (*f*_7_)	$f_{7}(x)=\overset{D}{\underset{i=1}{\sum }}\left|x_{i}\right|+\prod_{i=1}^{D}\left|x_{i}\right|$	50	[−500, 500]^*D*^	−837.9658
Alpine (*f*_8_)	$f(x)=\overset{D}{\underset{i=1}{\sum }}\left|x_{i}\sin\left(x_{i}\right)+0.1x_{i}\right|$	50	[−100, 100]^*D*^	0
Cross (*f*_9_)	$f_{10}(x)=\left|\sin\left(x_{1}\right)\sin(x_{2})e^{\left|100-\sqrt{x_{1}^{2}+x_{2}^{2}}/\pi \right|}+1\right|$	2	[−10, 10]^*D*^	0
Schaffer's F6 (*f*_10_)	$f_{9}(x)=0.5+\frac{\sin^{2}\left(\sqrt{x_{1}^{2}+x_{2}^{2}}\right)-0.5}{{\left(1+0.001(x_{1}^{2}+x_{2}^{2})\right)}^{2}}$	2	[−100, 100]^*D*^	0

**Table 2:** Results comparison of 10 test functions of 3 algorithms.

Function	Algorithm	Best	Worst	Mean	Std.
*f*_1_	ABC	8.85038*e* − 015	7.93808*e* − 005	2.65944*e* − 006	1.44905*e* − 005
	PABC	2.50765*e* − 015	4.22993*e* − 014	9.22438*e* − 015	8.65892*e* − 015
	IMABC	3.48561*e* − 017	8.9191*e* − 017	6.2547*e* − 017	1.33411*e* − 017

*f*_2_	ABC	5.90141	44.6327	18.4201	10.1225
	PABC	0.185485	7.19489	1.54921	1.72969
	IMABC	0.000204645	0.00921194	0.00381647	0.00285877

*f*_3_	ABC	4.44089*e* − 015	3.56306*e* − 009	1.32808*e* − 010	6.48804*e* − 010
	PABC	4.996*e* − 015	1.44799*e* − 010	7.14078*e* − 012	2.67773*e* − 011
	IMABC	0	0	0	0

*f*_4_	ABC	1.20019*e* − 008	11.6005	2.66884	2.94451
	PABC	7.42517*e* − 013	1.32277*e* − 005	5.01149*e* − 007	2.40813*e* − 006
	IMABC	0	0	0	0

*f*_5_	ABC	6.59917*e* − 013	1.04211*e* − 010	9.28022*e* − 012	1.86668*e* − 011
	PABC	4.21885*e* − 013	2.06368*e* − 011	4.90198*e* − 012	4.5731*e* − 012
	IMABC	8.88178*e* − 016	2.66454*e* − 015	2.54611*e* − 015	6.48634*e* − 016

*f*_6_	ABC	0.000957132	0.259845	0.0545307	0.0597714
	PABC	1.18297*e* − 013	3.51652*e* − 009	5.77724*e* − 010	9.57809*e* − 010
	IMABC	6.08383*e* − 021	1.4873*e* − 018	3.51197*e* − 019	2.97612*e* − 019

*f*_7_	ABC	−837.767	−826.26	−833.611	3.18261
	PABC	−730.356	−730.356	−730.356	3.46891*e* − 013
	IMABC	−837.966	−837.966	−837.966	0

*f*_8_	ABC	2.37327	6.15694	4.09479	1.21718
	PABC	0.0443285	0.419608	0.200788	0.142808
	IMABC	2.99468*e* − 016	5.48062*e* − 016	4.18311*e* − 016	8.39077*e* − 017

*f*_9_	ABC	4.85217*e* − 005	4.95895*e* − 005	4.87645*e* − 005	3.21009*e* − 007
	PABC	4.84822*e* − 005	4.86088*e* − 005	4.85033*e* − 005	3.7978*e* − 008
	IMABC	4.84822*e* − 005	4.84822*e* − 005	4.84822*e* − 005	4.03232*e* − 018

*f*_10_	ABC	0.000235347	0.0851231	0.0240707	0.0235249
	PABC	8.98791*e* − 011	8.1512*e* − 005	6.98519*e* − 006	1.63762*e* − 005
	IMABC	0	0	0	0
